# Vitamin D deficiency exacerbates bleomycin-induced pulmonary fibrosis partially through aggravating TGF-β/Smad2/3-mediated epithelial-mesenchymal transition

**DOI:** 10.1186/s12931-019-1232-6

**Published:** 2019-11-27

**Authors:** Se-Ruo Li, Zhu-Xia Tan, Yuan-Hua Chen, Biao Hu, Cheng Zhang, Hua Wang, Hui Zhao, De-Xiang Xu

**Affiliations:** 1 Second Affiliated Hospital, Anhui Medical University, Hefei, 230032 China; 2 Department of Toxicology, Anhui Medical University, 81 Meishan Road, Hefei, 230032 China

**Keywords:** Pulmonary fibrosis, Vitamin D deficiency, Epithelial-mesenchymal transition, Bleomycin, Mice

## Abstract

**Background:**

Our earlier report indicated that active vitamin D3 inhibited epithelial-mesenchymal transition (EMT) in bleomycin (BLM)-induced pulmonary fibrosis. The objective of this study was to further investigate whether vitamin D deficiency exacerbates BLM-induced pulmonary fibrosis.

**Methods:**

This study consists of two independent experiments. Experiment 1, male mice were fed with vitamin D deficient (VDD) fodder. Experiment 2, *Cyp27b1*^+/+^, *Cyp27b1*^+/−^ and *Cyp27b1*^−/−^ mice were fed with standard diet. For pulmonary fibrosis, mice were intratracheally instilled with a single dose of BLM (1.5 mg/kg). Serum 25(OH) D level was measured. Pulmonary collagen deposition was assessed by Sirius red staining. EMT was measured and transforming growth factor-beta (TGF-β)/Smad3 signaling was evaluated in the lungs of BLM-treated mice.

**Results:**

The relative weight of lungs was elevated in BLM-treated mice. Col1α1 and Col1α2, two collagen protein genes, were upregulated, and collagen deposition, as determined by Sirius red staining, was observed in the lungs of BLM-treated mice. E-cadherin, an epithelial marker, was downregulated. By contrast, vimentin and α-SMA, two EMT markers, were upregulated in the lungs of BLM-treated mice. Pulmonary TGF-β/Smad3 signaling was activated in BLM-induced lung fibrosis. Further analysis showed that feeding VDD diet, leading to vitamin D deficiency, aggravated elevation of BLM-induced relative lung weight. Moreover, feeding VDD diet aggravated BLM-induced TGF-β/Smad3 activation and subsequent EMT in the lungs. In addition, feeding VDD diet exacerbated BLM-induced pulmonary fibrosis. Additional experiment showed that *Cyp27b1* gene knockout, leading to active vitamin D3 deficiency, exacerbated BLM-induced pulmonary fibrosis. Moreover, *Cyp27b1* gene knockout aggravated pulmonary TGF-β/Smad2/3 activation and subsequent EMT in BLM-induced lung fibrosis.

**Conclusion:**

Vitamin D deficiency exacerbates BLM-induced pulmonary fibrosis partially through aggravating TGF-β/Smad2/3-mediated EMT in the lungs.

## Background

Idiopathic pulmonary fibrosis (IPF) has characteristics of fibroblast proliferation and collagen-based extracellular matrix reconstruction [1, 2]. The clinical manifestation of IPF includes progressive and irreversible loss of pulmonary function, ultimately causing respiratory failure [3]. Bleomycin (BLM), an antineoplastic agent, has been found to cause pulmonary interstitial fibrosis [4]. BLM-induced pulmonary fibrosis is characterized by extracellular matrix deposition, alveolar injury and inflammatory cell infiltration [5, 6]. Thus, BLM-induced pulmonary fibrosis has been used to mimics the process of IPF [7, 8]. Several studies have demonstrated that TGF-β-mediated epithelial-mesenchymal transition (EMT) plays an important role in the pathogenesis of BLM-induced pulmonary fibrosis [5, 9, 10].

Vitamin D is essential for uptake of calcium and metabolism of bone. The children with vitamin D deficiency have greater risks of rickets [11]. In the recent years, vitamin D is famous for its non-classical actions, such as its antioxidative and anti-inflammatory effects and its regulation of cell proliferation [12–15]. After hydroxylated by cytochrome P450 (CYP)2R1 to form 25(OH)D_3_, vitamin D_3_ is then transformed by CYP27B1 to form 1,25 (OH)_2_D_3_, the active form of vitamin D_3_. 1,25 (OH)_2_D_3_, exerts its biological function by activating vitamin D receptor (VDR) [16, 17]. Several animal experiments found that supplementation with active vitamin D_3_ alleviated BLM-induced pulmonary interstitial fibrosis [18, 19]. On the other hand, vitamin D deficiency or insufficiency is quite common especially in children and old people [20, 21]. Several epidemiological reports demonstrated that vitamin D deficiency was associated with increased risks of infections in lungs [22, 23]. According to a recent study, vitamin D deficiency was positively associated with the mortality of patients with IPF [24]. Moreover, this report found that supplementation with cholecalciferol protected mice from BLM-induced lung fibrosis [24]. Nevertheless, whether vitamin D deficiency exacerbates BLM-induced pulmonary fibrosis needs to be further determined.

In the present study, we established two mouse models of vitamin D deficiency through either feeding vitamin D deficient (VDD) diet or *Cyp27b1* gene knockout. The objective of this study was to investigate whether vitamin D deficiency aggravates BLM-induced pulmonary fibrosis using two different animal models. Our results indicated that both feeding VDD diet and *Cyp27b1* gene knockout exacerbated BLM-induced pulmonary fibrosis. Mechanistically, vitamin D deficiency exacerbated pulmonary fibrosis partially through aggravating BLM-induced TGF-β/Smad2/3-mediated EMT in the lungs.

## Material and methods

### Chemicals and reagents

Bleomycin (BLM) was from Hisun Pfizer Pharmaceuticals Co. Ltd. (Shanghai, China). 25(OH) D detection kit was from DIAsource (Louvain-la-Neuve, Belgium). TRIzol reagent was from Molecular Research Center, Inc. (Cincinnati, OH, USA). RNase-free DNase was purchased from Promega Corporation (Madison, WI, USA). ECL detection kit and Bicinchoninic acid (BCA) protein assay reagents were from Thermo Scientific (Massachusetts, MA, USA). Antibodies against Lamin A/C, β-actin, E-cadherin and vimentin were from Cell Signaling Technology (Danvers, MA, USA). Antibodies against α-SMA and Phospho-Smad3 were from Abcam (Cambridge, UK).

### Animals and treatments

The present study consists of two independent experiments. Experiment 1, male C57BL/6 J mice (4-week old) were from Beijing Vital River. All animals were kept with temperatures of 20–25 °C and humidity of 50%. All animals were fed standard diet and water freely. The circadian rhythm of 12 h light and 12 h darkness was maintained. After one week of adaptation, 40 mice were randomly divided into two groups: vitamin D deficient (VDD) and control (Ctrl). Mice of the VDD group were fed with vitamin D deficient fodder. Mice of the Ctrl group were fed with standard fodder. Five weeks later, mice of the Ctrl group and the VDD group were randomly divided into two subgroups, respectively (10 mice each subgroup): Ctrl, bleomycin (BLM), VDD, VDD + BLM. In the BLM and VDD + BLM groups, animals were intratracheally instilled with BLM (1.5 mg/kg). In the Ctrl and VDD groups, animals were treated with equal volume of saline in the same way. Experiment 2, wild-type (*Cyp27b1*^+/+^) and *Cyp27b1* knockout (*Cyp27b1*^−/−^) mice were from Beijing Vital River. Mating wild-type mice with *Cyp27b1* knockout mice produced *Cyp27b1*^+/+^, *Cyp27b1*^+/−^ and *Cyp27b1*^−/−^ mice. The genotype was confirmed by PCR analysis. All animals were fed with standard diet and water freely. The circadian rhythm of 12 h light and 12 h darkness was maintained. Six weeks after birth, mice each strain were randomly divided into 2 subgroups: Ctrl and BLM. In the BLM subgroup, mice were intratracheally instilled with BLM (1.5 mg/kg). In the Ctrl subgroup, mice were intratracheally instilled with equal volume of saline. All animals were sacrificed 2 weeks after BLM instillation. Serum 25(OH) D level was measured. The middle and lower lobes of right lung were sheared for histopathology and immunohistochemistry. The remainder tissues were collected for Western blots and real-time RT-PCR. The protocols of all animal experiments complied with the guidelines for humanitarian therapy by the Association of Laboratory Animal Sciences of Anhui Medical University and the Center for Laboratory Animal Sciences.

### Histology and determination of fibrosis

Lung tissue was fixed with 4% paraformaldehyde, embedded in paraffin. Each wax block was sliced continuously and stained with hematoxylin and eosin (H&E) to evaluate the histological manifestations. The inflammation was evaluated using the score of 0–5 according to previous study [19]. The numbers of inflammatory cells in every field (× 200) were counted. Lung fibrosis was determined by Sirius red staining according to our previous study with minor modification [5]. Briefly, after been rehydrated, the slices were stained with Sirius red for 1.5 h. Then rinsed the dye gently for about 3–5 s. The slices were washed with distilled water, gradient ethanol and xylene (× 2) each for 1 min. The percentage of collagen deposition was quantified using Image-Pro® Plus software. The data were shown as Mean ± SEM (*N* = 6).

### Immunohistochemistry

Lung was cut into sections (5 μm thick) according to the standard protocol [19, 25]. The slices were incubated with monoclonal antibodies against α-SMA (1000:1), ZEB1(200:1) and VDR (200:1) at 20–25 °C for 1 h and then 3–5 °C for 12 h (for α-SMA) or 18 h (for ZEB1 or VDR). After the color reaction, the slices were counterstained with hematoxylin. ZEB1- and VDR- positive nuclei and α-SMA-positive cells were counted at a magnification of × 400 (Leica, German). Fifteen visual fields were randomly selected for each slice.

### Western blotting

To extract total protein from lung tissue, 450 μl buffer with PMSF and 50 mg lung tissue were used. The supernate was collected after 35 min grinding on ice and centrifugated for 15 min. The protein concentration of total pulmonary lysate was measured with the BCA protein assay reagents. In order to extract nuclear protein from lung tissue, 300 mg lung tissue was homogenized with 4 ml buffer A [25]. Supernate was gleaned after centrifugation for 40 s at 700 rpm. The mixed precipitate was gleaned after supernate was centrifuged for 25 min at 6500 rpm. Mixed precipitate was suspended in 105 μl lysis buffer B [25]. After maintaining a static position at 4 °C for 60 min, solution was stirred with electric stirring rod for 4 min. Supernate was gleaned after centrifugation for 10 min at 12000 rpm. The protein concentration of nuclear lysate was measured with the BCA protein assay reagents. For Western blotting, equivalent protein (8~22 μg) was separated by 10% SDS-PAGE and transferred to a polyvinylidene fluoride membrane. For total protein, membranes were incubated for 1~2 h using following antibodies: E- cadherin, vimentin, α-SMA, p-Smad3, or β-actin. For nucleoprotein, membranes were incubated with either VDR antibody for 18 h or Lamin A/C antibody for 12 h at 3–5 °C. After the membranes were washed for four times, second antibody was used to incubate for 1~2 h. Finally, signal was measured by ECL kit.

### Isolation of Total RNA and real-time PCR

TRIzol reagent was used to collect total RNA from mouse lungs. After precipitating and washing, total RNA was determined by OD value to quantity as 0.5 μg/μl. RNA was then reverse-transcribed with AMV after digestion with RNA-free DNase. Specific primers (Table 1) and GoTaq1 qPCR master mix were used to carry out real-time PCR. The amplified reaction program was performed with the Light Cycler 480 Instrument. The specific process was referred to our previous experiments [19].

**Table 1:** Primers for RT-PCR

Genes	Forward (5′ -3′)	Reverse (5′ -3′)
*18 s*	GTAACCCGTTGAACCCCATT	CCATCCAATCGGTAGTAGCG
*Col1α1*	CAATGGCACGGCTGTGTGCG	AGCACTCGCCCTCCCGTCTT
*Col1α2*	CTCATACAGCCGCGCCCAGG	AGCAGGCGCATGAAGGCGAG
*Tgf-β1*	TTCCGCTGCTACTGCAAGTCA	GGGTAGCGATCGAGTGTCCA

### Statistics

The raw data were presented as means ± S.E.M. ANOVA was used to calculate differences between different groups. All data were evaluated for significance using non-parametric tests techniques when those were not normally distributed. *P* < 0.05 and *P* < 0.01 were determined to demonstrate statistically significant. All statistical analyses were carried out with SPSS 19.0 software.

## Results

### Vitamin D deficiency aggravates BLM-induced pulmonary inflammation and interstitial fibrosis

**Fig. 1:**
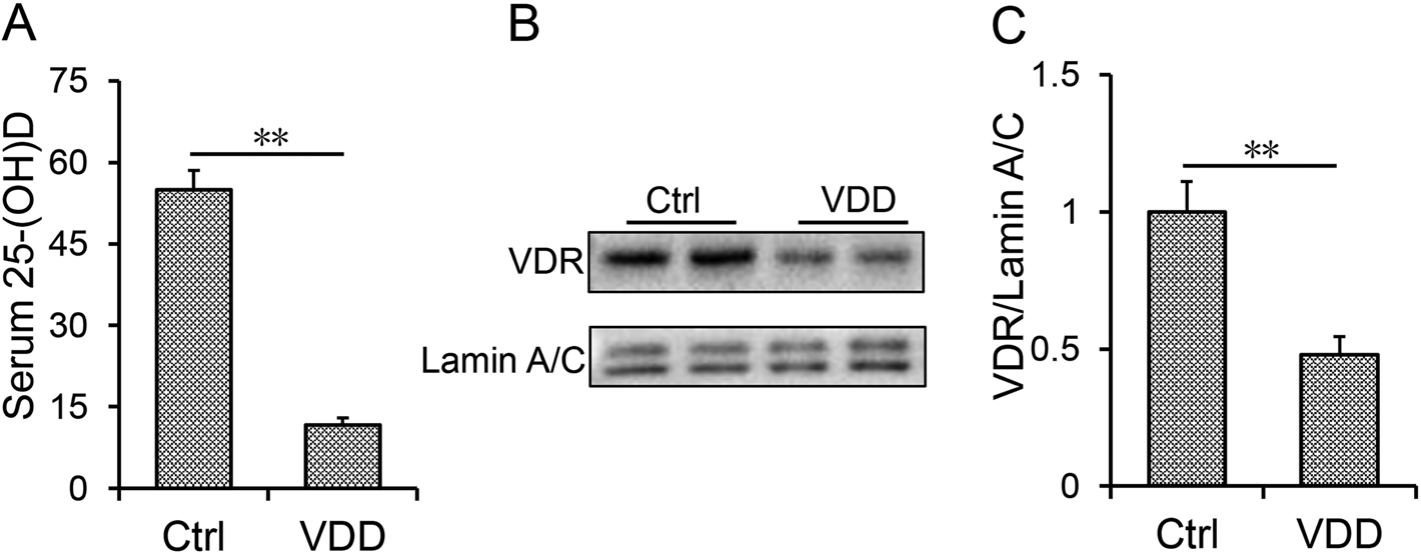
Serum 25 (OH) D and nuclear VDR level. During the VDD group, animals were fed with vitamin D deficient fodder. During the Ctrl group, animals were fed with standard fodder. All animals except controls were injected with BLM (1.5 mg/kg) through the trachea. All animals were sacrificed 2 weeks after BLM. **a** Serum 25(OH) D concentrations were estimated using RIA. **b** and **c** Nuclear VDR was estimated using Western blot. All experiments were repeated for 3 times. Data were expressed as means ± SEM (*N* = 6). ** *P* < 0.01

**Fig. 2:**
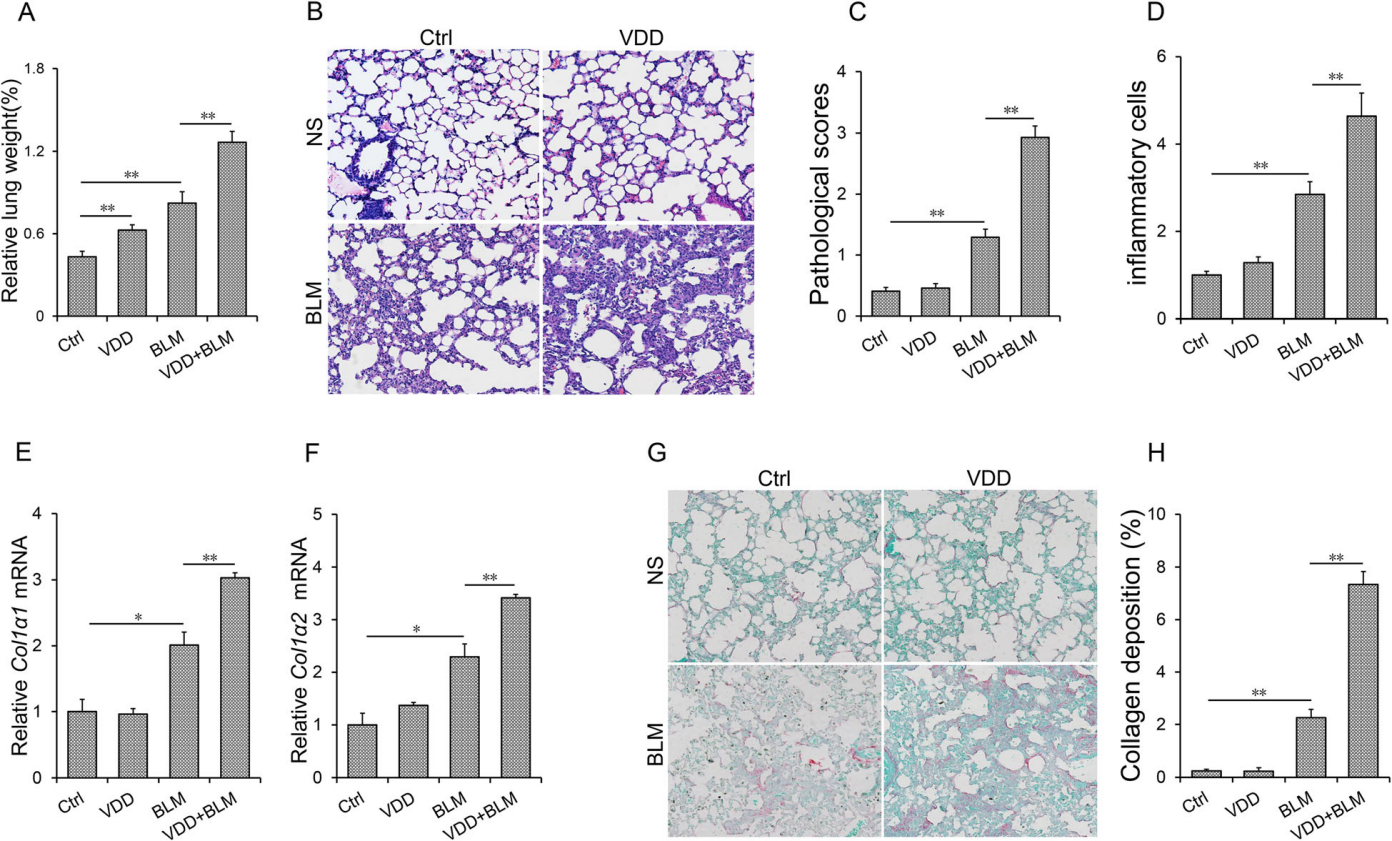
Vitamin D deficiency aggravates BLM-induced pulmonary inflammation and interstitial fibrosis*.* In the VDD group, animals were fed with vitamin D deficient fodder. In the Ctrl group, animals were fed with standard fodder. All animals except controls were injected with BLM (1.5 mg/kg) through the trachea. All animals were sacrificed 2 weeks after BLM. **a** Relative lung weight was measured. **b**-**d** Pulmonary histopathological injury and inflammation were evaluated by H&E. **b** A representative picture. Original magnification: 200×. **c** Pulmonary pathological scores. **d** Inflammatory cells in the lungs. **e**-**f** Col1α1 and Col1α2 were estimated with real-time PCR. (G-H) Collagen deposition was evaluated using Sirius red staining. **g** A representative picture. Original magnification: 200×. **h** The area of collagen deposition was calculated. All data were expressed as means ± SEM (*N* = 6). **P < 0.01, *P < 0.05

Serum 25(OH) D concentrations were evaluated. It showed that serum 25 (OH) D concentration was significantly lower in VDD group than that of control group (Fig. 1a). Nuclear VDR level of lungs was accordingly reduced among VDD-fed mice (Fig. 1b and c). As shown in Fig. 2a, the relative weight of lungs was slightly increased in BLM-treated group. Numerous inflammatory cells were infiltrated in lungs of mice injected with BLM, accompanied by collapse of lung tissue and thickening of alveolar septum (Fig. 2b). Of interest, the relative weight of lungs was higher in VDD + BLM group than that of BLM group (Fig. 2a). Moreover, BLM-induced infiltration of inflammatory cells and pathological damage were aggravated in VDD diet fed mice (Fig. 2b-d). To further evaluate BLM-induced pulmonary fibrosis, pulmonary collagen (*Col1α1* and *Col1α2*) mRNAs were measured by real-time RT-PCR. As expected, pulmonary *Col1α1* and *Col1α2* mRNAs were obviously upregulated in BLM-treated mice (Fig. 2e-f). Sirius red staining was performed for lung fibrosis. As shown in Fig. 2g and h, collagen deposition was discovered in the lungs in BLM-treated mice. Interestingly, BLM-induced upregulation of *Col1α1* and *Col1α2* mRNAs was aggravated in the lungs of VDD diet-fed mice (Fig. 2e and f). Correspondingly, BLM-induced collagen deposition, as determined by Sirius red staining, was aggravated in the lungs of VDD diet-fed mice (Fig. 2g and h, 2.27 ± 0.32 VS 7.34 ± 0.49, *P* < 0.01).

### Vitamin D deficiency aggravated BLM-induced EMT in the lungs

**Fig. 3:**
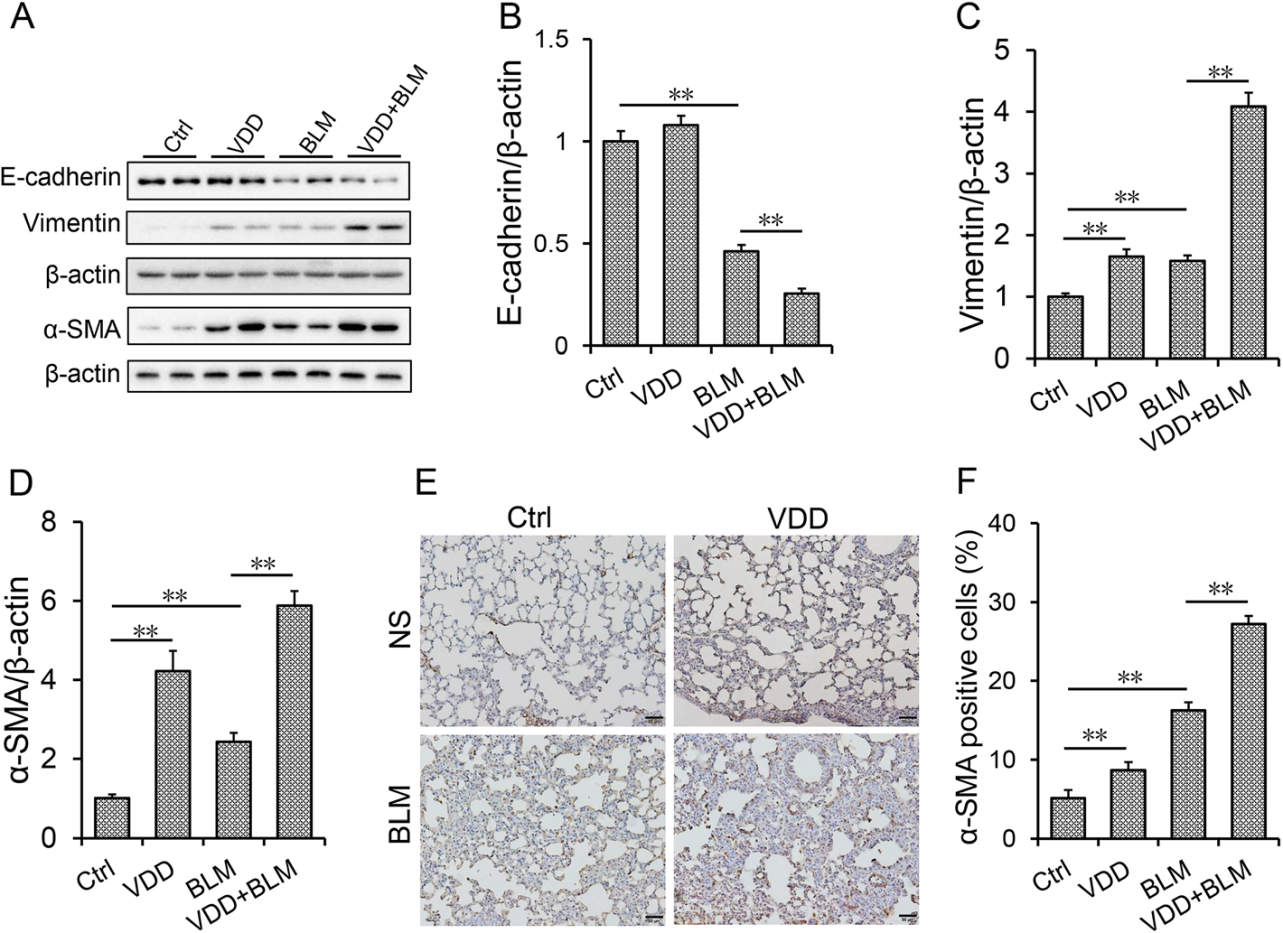
Vitamin D deficiency aggravates BLM-induced EMT in the lungs. During the VDD group, animals were fed with vitamin D deficient fodder. During the Ctrl group, animals were fed with standard fodder. All animals except controls were injected with BLM (1.5 mg/kg) through the trachea. All animals were sacrificed 2 weeks after BLM. **a**-**d** Pulmonary E-cadherin, vimentin and α-SMA were detected using Western blot. **a** Representative gels for E-cadherin, vimentin and α-SMA and β-actin. Quantitative analyses of scanning densitometry for **b** E-cadherin, **c** vimentin and **d** α-SMA were performed. **e** Pulmonary α-SMA was measured using IHC. Original magnification: 200×. **f** α-SMA-positive cells were calculated. All data were expressed as means ± S.E.M. (*N* = 6). **P < 0.01, *P < 0.05

The influences of vitamin D deficiency on BLM-induced EMT were evaluated. It showed that, pulmonary E-cadherin, an epithelial marker, was obviously downregulated in BLM-treated group (Fig. 3a and b). By contrast, pulmonary vimentin, a mesothelial marker, was slightly upregulated in BLM-treated mice (Fig. 3a and c). Although it had little effect on the expression of E-cadherin, vitamin D deficiency aggravated BLM-induced downregulation of E-cadherin in the lungs (Fig. 3a and b). Unexpectedly, vitamin D deficiency slightly upregulated the expression of pulmonary vimentin. Moreover, vitamin D deficiency aggravated BLM-induced upregulation of vimentin in the lungs (Fig. 3a and c). The effects of vitamin D deficiency on α-SMA, another EMT marker, were detected. As shown in Fig. 3a and d, pulmonary α-SMA was significantly upregulated in BLM-treated mice. IHC exhibited that α-SMA-positive cells were significantly increased in the lungs of mice treated with BLM (Fig. 3e-f). Unexpectedly, vitamin D deficiency upregulated the expression of pulmonary α-SMA (Fig. 3a, d-f). Moreover, vitamin D deficiency aggravated BLM-induced upregulation of α-SMA in the lung (Fig. 3a, d-f).

### Vitamin D deficiency aggravates BLM-induced TGF-β/Smad3 activation in the lungs

**Fig. 4:**
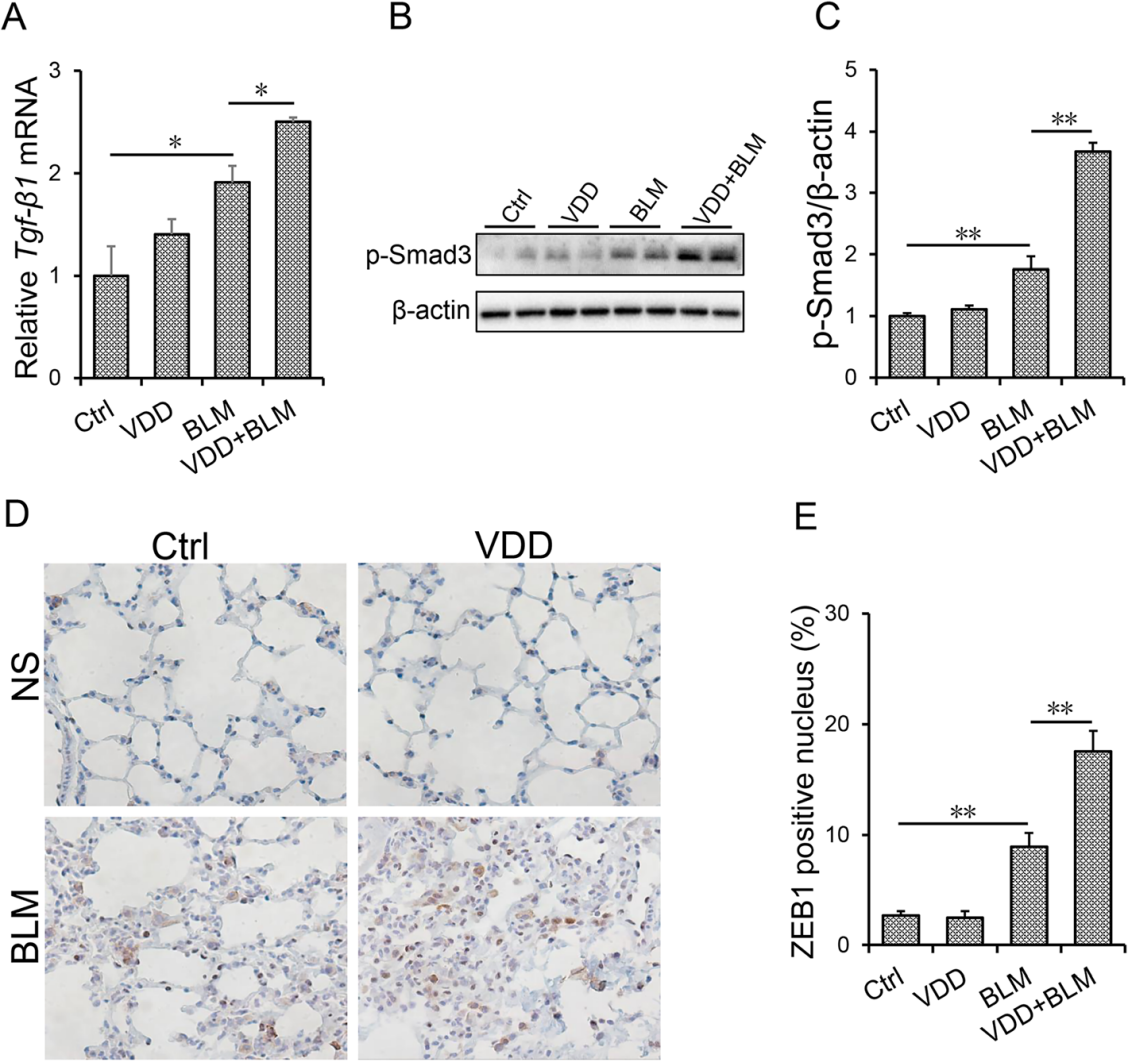
Vitamin D deficiency aggravates BLM-induced activation of TGF-β/Smad3 in the lungs. During the VDD group, animals were fed with vitamin D deficient fodder. During the Ctrl group, animals were fed with standard fodder. All animals except controls were injected with BLM (1.5 mg/kg) through the trachea. All animals were sacrificed 2 weeks after BLM. **a** Pulmonary *Tgf-β1* mRNA was measured by real-time RT-PCR. **b** and **c** Phosphorylated Smad3 was detected using Western blot. Quantitative analysis of scanning densitometry for p-Smad3 was performed. **d** and **e** Pulmonary ZEB1 was measured using IHC. **d** A representative picture. Original magnification: 200×. **e** ZEB1-positive cells were calculated. All data were expressed as means ± SEM (N = 6). **P < 0.01, *P < 0.05

As expected, the expression of pulmonary *Tgf-β1* mRNA was significantly upregulated in BLM-treated mice (Fig. 4a). The level of pulmonary p-Smad3 was accordingly elevated in BLM-treated mice (Fig. 4b and c). ZEB1, a downstream molecule of TGF-β/Smad3 signaling, was determined using IHC. As shown in Fig. 4d and e, ZEB1-positive cells were obviously elevated in BLM-treated mice. The effects of vitamin D deficiency on BLM-induced activation of TGF-β/Smad3 signaling were then analyzed. Of interest, vitamin D deficiency aggravated BLM-induced upregulation of *Tgf-β1* mRNA in the lung (Fig. 4a). Moreover, vitamin D deficiency aggravated BLM-induced pulmonary Smad3 phosphorylation (Fig. 4b and c). In addition, vitamin D deficiency aggravated BLM-induced elevation of ZEB1-positive cells in the lungs (Fig. 4d and e).

### Cyp27b1 gene knockout aggravates BLM-induced pulmonary inflammation and interstitial fibrosis

**Fig. 5:**
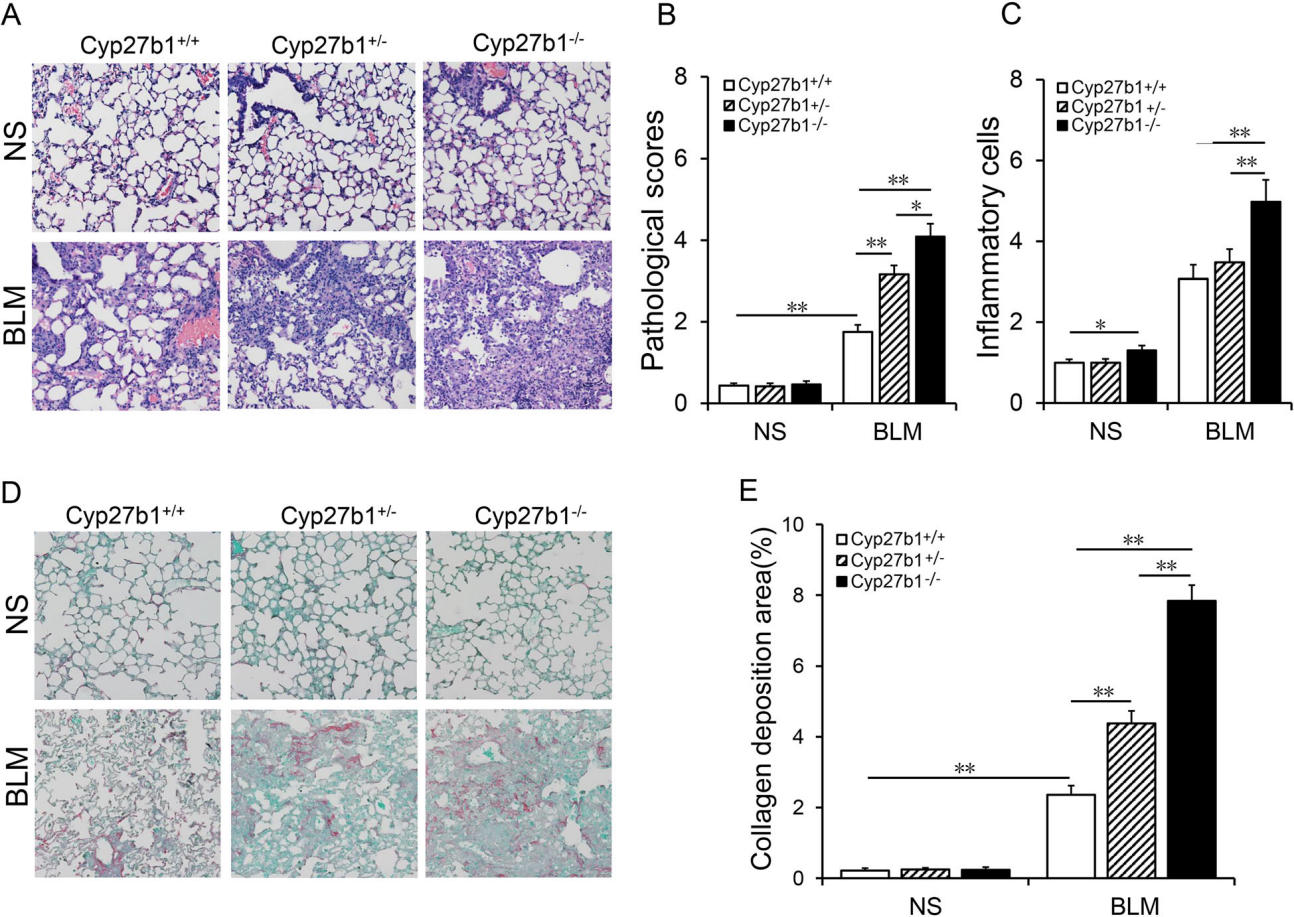
*Cyp27b1* gene knockout aggravates BLM-induced pulmonary interstitial fibrosis. All animals were fed with standard fodder. All animals except controls were injected with BLM (1.5 mg/kg) through the trachea. All animals were sacrificed 2 weeks after BLM. **a** and **b** Pulmonary histopathological injury and inflammation were evaluated by H&E. **a** A representative picture. Original magnification: 200×. **b** Pulmonary pathological scores were evaluated. **c** Inflammatory cells in the lungs. **d** and **e** Collagen deposition was evaluated using Sirius red staining. **d** A representative picture. Original magnification: 200×. **e** The area of collagen deposition was calculated. All data were expressed as means ± SEM (N = 6). **P < 0.01, *P < 0.05

As shown in Fig. 5a, numerous inflammatory cells were infiltrated in lungs of *Cyp27b1* wild-type mice injected with BLM, accompanied by collapse of lung tissue and thickening of alveolar septum. Of interest, pathological scores were higher in BLM-treated *Cyp27b1*^*+/−*^ mice than in BLM-treated *Cyp27b1* wild-type mice, with the highest pathological scores in BLM-treated *Cyp27b1*^*−/−*^ mice (Fig. 5b). Moreover, BLM-induced the infiltration of inflammatory cells was aggravated in *Cyp27b1* gene knockout mice (Fig. 5c). Pulmonary collagen deposition was determined using Sirius red staining. As shown in Fig. 5d, collagen deposition was discovered in the lungs of BLM-treated *Cyp27b1* wild-type mice. Interestingly, the area of collagen deposition was larger in BLM-treated *Cyp27b1*^*+/−*^ mice (4.38 ± 0.35) than that of BLM-treated *Cyp27b1* wild-type mice (2.36 ± 0.27), with the most collagen deposition in BLM-treated Cyp27b1^−/−^ mice (7.83 ± 0.45) (Fig. 5d-e, F = 58.868, *P* < 0.01).

### Cyp27b1 gene knockout aggravates BLM-induced EMT in the lungs

**Fig. 6:**
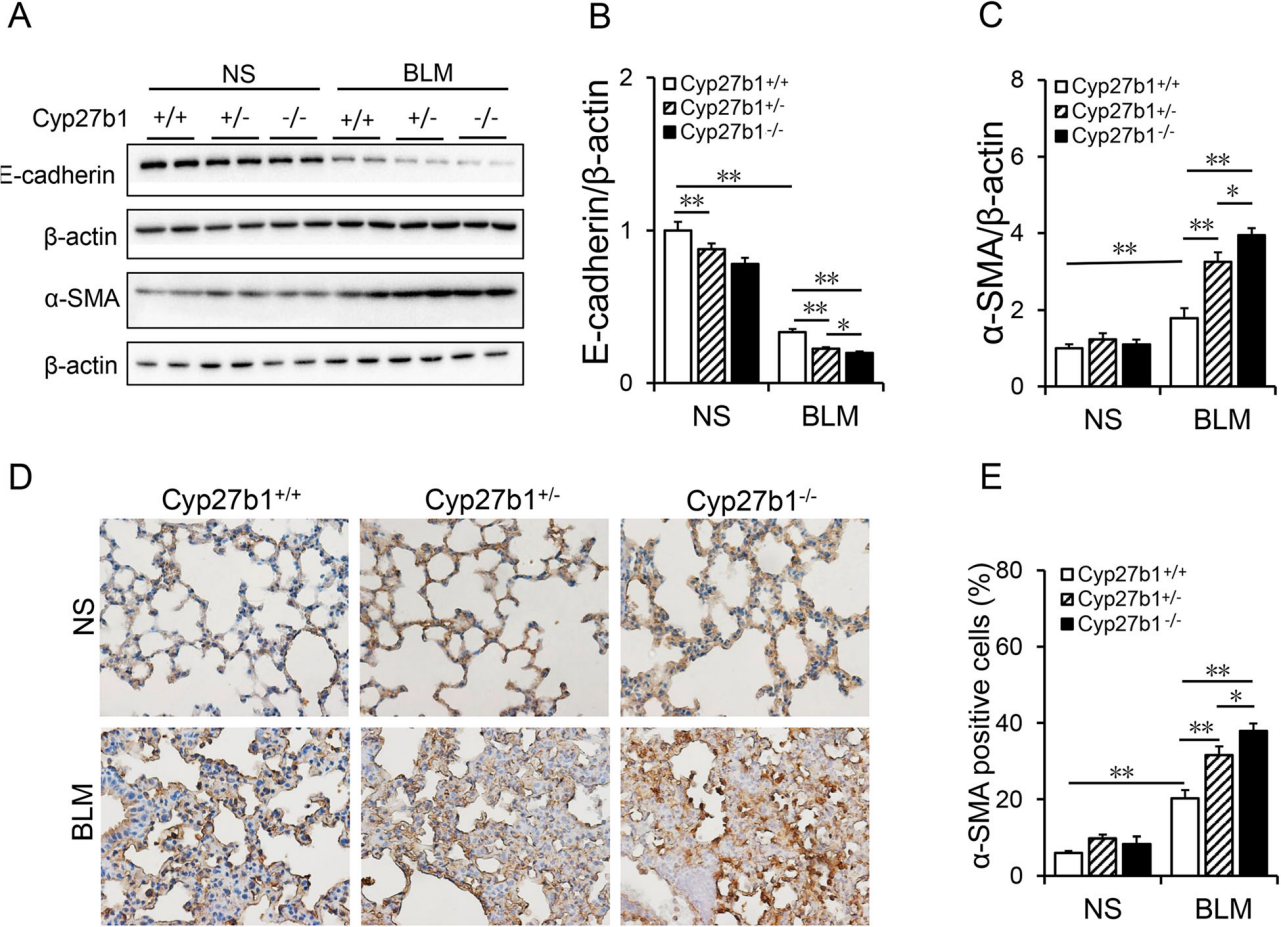
*Cyp27b1* gene knockout aggravates BLM-induced EMT. All animals were fed with standard fodder. All animals except controls were injected with BLM (1.5 mg/kg) through the trachea. All animals were sacrificed 2 weeks after BLM. **a**-**c** Pulmonary E-cadherin and α-SMA were detected using Western blot. **a** Representative gels for E-cadherin, α-SMA and β-actin. Quantitative analyses of scanning densitometry for **b** E-cadherin and **c** α-SMA were performed. **d** Pulmonary α-SMA was measured using IHC. Original magnification: 200×. **e** α-SMA-positive cells were calculated. All data were expressed as means ± S.E.M. (N = 6). **P < 0.01, *P < 0.05

The effects of *Cyp27b1* gene knockout on BLM-induced EMT were evaluated. As expected, pulmonary E-cadherin, an epithelial marker, was obviously downregulated in BLM-treated *Cyp27b1* wild-type mice (Fig. 6a and b). By contrast, pulmonary α-SMA, a mesothelial marker, was upregulated in BLM-treated *Cyp27b1* wild-type mice (Fig. 6a and c). Interestingly, pulmonary E-cadherin was downregulated in *Cyp27b1*^*−/−*^ mice. Moreover, *Cyp27b1* knockout aggravated BLM-induced downregulation of E-cadherin in the lungs (Fig. 6a and b). Although it had little effect on pulmonary α-SMA, *Cyp27b1* knockout aggravated BLM-induced downregulation of α-SMA in the lungs (Fig. 6a and c). IHC showed that α-SMA-positive cells were elevated in the lungs of *Cyp27b1* wild-type mice treated with BLM (Fig. 6d and e). Unexpectedly, *Cyp27b1* knockout upregulated pulmonary α-SMA. Moreover, *Cyp27b1* knockout aggravated BLM-induced upregulation of α-SMA in the lung (Fig. 6d-e).

### Cyp27b1 gene knockout aggravates BLM-induced TGF-β/Smad3 activation in the lungs

**Fig. 7:**
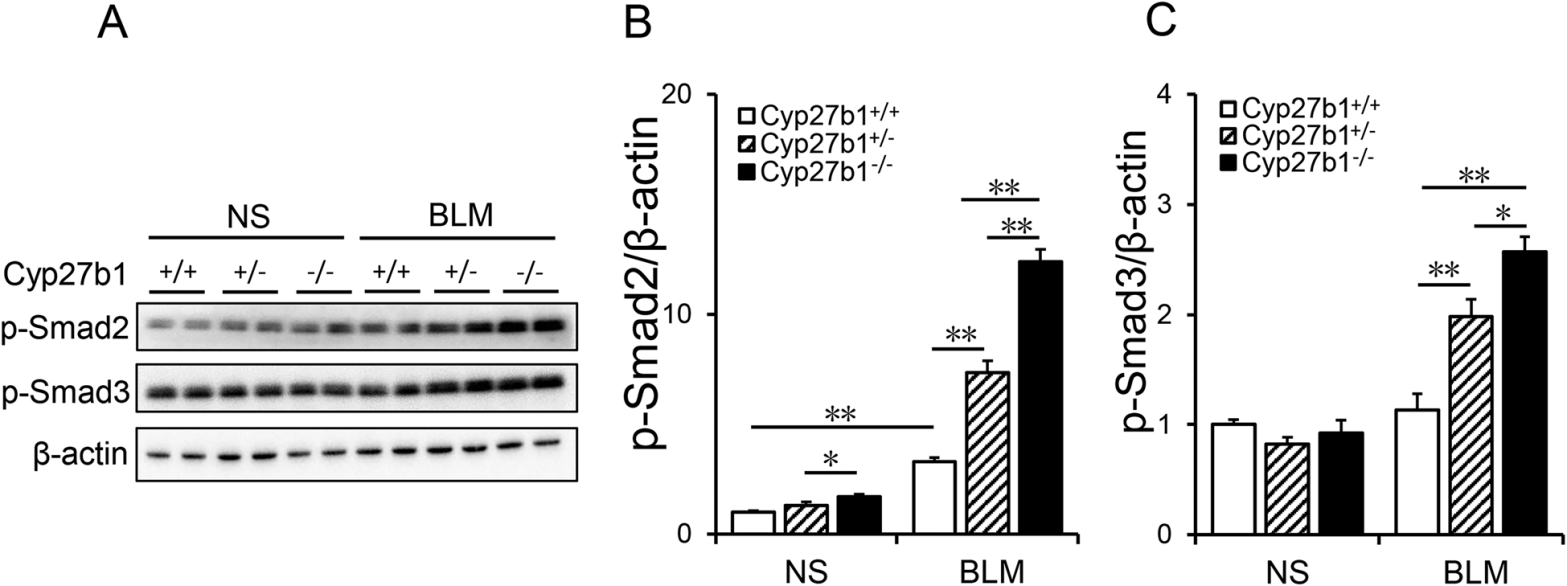
*Cyp27b1* gene knockout aggravates BLM-induced activation of TGF-β/Smad3 in the lungs. All animals were fed with standard fodder. All animals except controls were injected with BLM (1.5 mg/kg) through the trachea. All animals were sacrificed 2 weeks after BLM. Phosphorylated Smad2 and Smad3 were detected using Western blot. **a** Representative gels for p-Smad2, p-Smad3 and β-actin. Quantitative analysis of scanning densitometry for **b** p-Smad2 and **c** p-Smad3 were performed. Data were expressed as means ± SEM (N = 6). **P < 0.01, *P < 0.05

As expected, pulmonary p-Smad2 and p-Smad3 were elevated in BLM-treated *Cyp27b1* wild-type mice (Fig. 7a-c). The effects of *Cyp27b1* gene knockout on BLM-induced activation of Smad2/3 pathway were then analyzed. Of interest, *Cyp27b1* gene knockout aggravated BLM-induced Smad2 and Smad3 phosphorylation in the lungs (Fig. 7a-c).

## Discussion

In the present study, we investigated the effects of feeding VDD diet, which resulted in vitamin D deficiency, on BLM-induced pulmonary fibrosis. The data showed that vitamin D deficiency exacerbated BLM-induced interstitial inflammation in the lungs. Moreover, vitamin D deficiency aggravated BLM-induced upregulation of pulmonary *Col1α1* and *Col1α2* mRNAs. In addition, vitamin D deficiency aggravated BLM-induced collagen deposition in the lungs, as determined by Sirius red staining. Results above exhibit the first experimental evidence that vitamin D deficiency exacerbates BLM-induced pulmonary fibrosis.

Accumulating data have demonstrated that TGF-β/Smad2/3-mediated EMT plays an important role in the development of BLM-induced pulmonary fibrosis [26, 27]. Indeed, our study showed that pulmonary TGF-β/Smad2/3 signaling was activated during BLM-induced lung fibrosis. Pulmonary E-cadherin, an epithelial marker, was obviously downregulated in BLM-treated mice. By contrast, vimentin and α-SMA, two mesothelial markers, were upregulated in the lungs of BLM-treated mice. In addition, numerous α-SMA-positive cells, as determined by IHC, were observed in pulmonary interstitium. An earlier in vitro study indicated that active vitamin D3 inhibited the expression of α-SMA and prevented the upregulation of fibronectin and collagen in TGF-β1-stimulated fibroblasts [28]. Recently, two reports indicated that calcitriol, the active form of vitamin D3, suppressed TGF-β1-stimulated EMT in human bronchial epithelial cells [29, 30]. In the present study, we investigated whether vitamin D deficiency aggravates Smad2/3-mediated EMT in BLM-induced lung fibrosis. Our results showed that vitamin D deficiency aggravated pulmonary TGF-β1 upregulation and subsequent Smad3 phosphorylation in BLM-induced lung fibrosis. Moreover, vitamin D deficiency aggravated upregulation of pulmonary ZEB1, a transcription factor for EMT, during BLM-induced lung fibrosis. Finally, vitamin D deficiency aggravated BLM-induced downregulation of pulmonary E-cadherin. By contrast, vitamin D deficiency aggravated BLM-induced upregulation of vimentin and α-SMA in the lungs. These results suggest that vitamin D deficiency exacerbates BLM-induced pulmonary fibrosis, at least partially, through aggravating TGF-β/Smad2/3-mediated EMT.

*Cyp27b1* gene encodes CYP27B1, which is responsible for converting 25(OH)D_3_ into 1,25(OH)_2_D_3_, an active form of vitamin D_3_ [31, 32]. So *Cyp27b1* gene knockout results in active vitamin D_3_ deficiency [33, 34]. An earlier study indicated that active vitamin D_3_ deficiency exacerbated radiation-induced bone marrow injury [35]. In the present study, we observed the effects of active vitamin D_3_ deficiency on BLM-evoked EMT and pulmonary fibrosis using a model of *Cyp27b1* gene knockout mice. Of interest, *Cyp27b1* knockout aggravated collagen deposition, as accessed by Sirius red staining, in BLM-induced pulmonary fibrosis. In addition, *Cyp27b1* knockout aggravated BLM-induced downregulation of E-cadherin in the lungs. By contrast, *Cyp27b1* knockout aggravated BLM-induced upregulation of α-SMA in the lungs. Finally, *Cyp27b1* gene knockout aggravated BLM-induced pulmonary Smad2 and Smad3 phosphorylation. These results provide additional evidence that active vitamin D_3_ deficiency exacerbates Smad2/3 activation and subsequent EMT in BLM-induced pulmonary fibrosis.

The mechanism through which vitamin D deficiency aggravates TGF-β/Smad2/3-mediated EMT during BLM-induced pulmonary fibrosis remains obscure. An earlier report showed that active vitamin D3 inhibited renal fibrosis through promoting direct interaction VDR and Smad3 [36]. A recent study found that VDR, as a negative regulator of TGF-β/Smad3 pathway, inhibited Smad3-dependent transcription through stimulating formation of complexes between VDR and phosphorylated Smad3 in fibroblasts [37]. Indeed, the present experiment showed that pulmonary VDR signaling was inactivated in VDD-fed mice. Thus, it is speculated that VDR may be a regulator of EMT during BLM-induced pulmonary fibrosis. Indeed, an earlier report claimed that the truncated E-cadherin was elevated in biliary epithelial cells silenced for VDR [38]. Another study found that the expression of E-cadherin was downregulated in gut epithelial cells from VDR knockout mice [39]. In our present study, we claimed that pulmonary vimentin and α-SMA, two mesothelial markers, was upregulated in VDD-fed mice. By contrast, pulmonary E-cadherin, an epithelial marker, was slightly downregulated in *Cyp27b1* gene knockout mice. Therefore, our present study does not exclude the direct role of VDR on EMT. Further study is necessary to explore whether long-term vitamin D deficiency induces pulmonary fibrosis through directly regulating EMT in the lungs.

VDR as a regulator of EMT during BLM-induced pulmonary fibrosis may have preventive and therapeutic implications. According to an epidemiological report, vitamin D deficiency was positively associated with the mortality of patients with IPF [24]. A recent animal experiment found that supplementation with calcitriol, an active vitamin D3, suppressed interstitial inflammation and EMT in the pathogenesis of BLM-induced lung fibrosis [19]. Another animal experiment indicated that supplementation with cholecalciferol, another active vitamin D3 protected mice from BLM-induced EMT and subsequent lung fibrosis [24]. In the present study, we showed that vitamin D deficiency aggravated BLM-induced TGF-β/Smad2/3 activation and EMT in the lungs. In addition, vitamin D deficiency exacerbated BLM-induced pulmonary fibrosis. Thus, vitamin D3 may be used as a potential adjuvant agent for pulmonary fibrosis especially in patients with low vitamin D status.

## Conclusion

In summary, our present experiment investigated the effects of vitamin D deficiency on BLM-induced pulmonary fibrosis using two mouse models. We showed that feeding VDD diet, leading to vitamin D deficiency, exacerbated BLM-induced pulmonary fibrosis. Moreover, vitamin D deficiency aggravated TGF-β/Smad2/3 activation and subsequent EMT during BLM-induced pulmonary fibrosis. We found that *Cyp27b1* gene knockout, leading to active vitamin D3 deficiency, exacerbated BLM-induced pulmonary fibrosis. In addition, *Cyp27b1* gene knockout aggravated pulmonary TGF-β/Smad2/3 activation and subsequent EMT during BLM-induced lung fibrosis. Data above provide evidence that vitamin D deficiency exacerbates BLM-induced pulmonary fibrosis partially through aggravating TGF-β/Smad2/3-mediated EMT in the lungs.

## Data Availability

Data sharing is not applicable to this article as no datasets were generated or analyzed during the present study.

## Abbreviations

BLM: Bleomycin
CYP: Cytochrome P450
EMT: Epithelial–mesenchymal transition
IHC: Immunohistochemistry
IPF: Idiopathic pulmonary fibrosis
RT-PCR: Reverse transcription-polymerase chain reaction
Transforming TGF-β: Growth factor-beta
VDD: Vitamin D deficient
VDR: Vitamin D receptor
α-SMA: alpha smooth muscle actin
